# Cancer treatment-related financial toxicity in Japan: a scoping review

**DOI:** 10.3389/fpsyg.2023.1205016

**Published:** 2023-08-01

**Authors:** Yuki Itani, Kyoko Obama, Maiko Fujimori, Junko Saito, Yosuke Uchitomi

**Affiliations:** ^1^Division of Survivorship Research, National Cancer Center Institute for Cancer Control, Tokyo, Japan; ^2^Division of Behavioral Sciences, National Cancer Center Institute for Cancer Control, Tokyo, Japan

**Keywords:** cancer treatment, financial toxicity, cancer survivorship, comprehensive score for financial toxicity, financial burden

## Abstract

Financial toxicity during cancer survival has been studied mainly in the United States; 47–49% of cancer survivors reported financial hardships and 12–63% reported debt owing to treatment costs. Financial toxicity is influenced by each country’s economic status and healthcare system. We aimed to review the evidence on financial toxicity in Japan. A systematic search was performed using PubMed and Ichushi databases. We included English or Japanese peer-reviewed articles that (1) explored the experiences of cancer patients facing financial toxicity due to cancer diagnosis and treatment, (2) were specific to Japan, and (3) focused on the experiences of financial toxicities among cancer patients. Data were extracted focusing on the experiences of patients, families, and healthcare providers. The main themes were synthesized based on a previous study. The search yielded 632 citations from PubMed and 21 from Ichushi, and non-duplicates were identified. Of these, 31 articles were selected for full-text review. Literature was divided into studies describing the following elements: (a) risk factors for financial toxicity, (b) description of financial toxicity, (c) psychological reactions, (d) coping strategies for financial toxicity, and (e) impact on treatment outcomes. Only three studies reported comprehensive financial toxicity scores. Furthermore, treatment costs influenced physicians’ treatment decisions, and patients and their families adopted various strategies to cope with treatment costs. Two studies showed that low current income and younger age were high-risk factors. As for utilization of the support system, approximately 70% of the patients used the high-cost medical expense system, 20% used the sickness benefit system, and 40% used the medical expense deduction system. Many cancer patients in Japan suffer from financial toxicity during cancer survival. One reason for this is that the awareness of the system supporting financial toxicity is insufficient and actual utilization is low. It is necessary to actively encourage patients to ask healthcare providers questions, improve the link between patients and the support system, reconstruct the support system design, and improve the method of publicizing the system.

## 1. Introduction

In Japan, one million new cancer cases occur annually (Cancer registry and statistics, 2017). For patients and their families, an existential issue that arises with a cancer diagnosis is the financial burden. The highest average medical expenses over five years for cancer treatment were for esophageal cancer (5,677,000 yen), followed by colorectal cancer (4,438,000 yen) and hepatobiliary-pancreatic cancer (4,473,000 yen). Patients with cancer are likely to experience greater financial toxicity than people without cancer (Guy et al., 2017). The increasing number of long-term cancer survivors has led to an increased focus on financial toxicity.

“Financial toxicity” is a term widely used to describe the material and emotional burden experienced by patients during and after cancer treatment due to financial hardships (Hussaini et al., 2022; PDQ® Adult Treatment Editorial Board, 2022). Previous studies have shown that material hardships, psychological responses, and coping behaviors are getting more research attention (Witte et al., 2019; Udayakumar et al., 2022). Financial toxicity results from the material, psychological, and behavioral burden experienced by cancer patients, their families, and healthcare providers as a result of cancer diagnosis. Although no standardized taxonomy exists, material burden means an increase in expenses and a change in employment, psychological burden includes anxiety about medical expenses and concerns about treatment and treatment costs, and behavioral burden refers to the impact of treatment costs on patients, families, and healthcare providers behaviors, as well as treatment adherence of patients and supportive behaviors of healthcare providers (Udayakumar et al., 2022). A systematic review of the risk factors and outcomes of financial toxicity from cancer treatment in the United States (Smith et al., 2019) found that 49% of approximately 600,000 patients across 74 studies reported material and psychological burdens. Socioeconomic predictors of an exacerbated economic burden include lack of health insurance, lower income, loss of employment, and younger age at cancer diagnosis (Smith et al., 2019). Multifaceted studies of financial toxicity have been conducted worldwide, including in low- and middle-income countries (Udayakumar et al., 2022).

As material burden includes direct costs, such as medicine and hospitalization costs, and indirect costs, such as transportation and food costs, different consequences regarding material burden would occur in Japan, where a publicly funded healthcare system is adopted. Systematic reviews have indicated that even in countries with publicly funded universal coverage, there is a material burden because public financial support is not sufficient to address the increased out-of-pocket payments and decreased income associated with diagnosis (Longo et al., 2020; Fitch et al., 2022). Indeed, several studies have reported that material burden occurs even in Japan, where universal health coverage has been achieved (Takahashi, 2016; Honda, 2018). However, the evidence is still limited and not summarized systematically to inform policy guidelines or intervention development.

Therefore, we conducted a scoping review to describe a wide range of studies on financial toxicity in Japan, including current prevalence, coping strategies, and the impact on treatment outcomes. The findings of this scoping review can be used to gain insight into knowledge gaps and help focus future research and policymaking toward improving financial toxicity associated with a cancer diagnosis. We aimed to provide an overview of financial toxicity experienced by cancer patients, their families, and healthcare providers in Japan. We focused on describing the entire body of evidence to answer the following question: What is known in the available literature about the financial toxicity of patients undergoing cancer diagnosis and treatment in Japan, including their families and healthcare professionals, specifically about the risk factors and impact of financial toxicity, particularly material burden, psychological reactions, and behavioral outcomes?

## 2. Materials and methods

We used the latest scoping review framework suggested by the Joanna Briggs Institute (JBI) (Peters et al., 2020), which includes (1) identifying the research question; (2) recognizing relevant studies; (3) choosing studies; (4) charting the data; and (5) collating, synthesizing, and reporting the results. Our scoping review followed the Preferred Reporting Items for Systematic Reviews and Meta-Analyses extension for Scoping Reviews (PRISMA-ScR) guidelines (Tricco et al., 2018). We drafted a protocol to ensure accurate, unbiased, and comprehensive compilation and analysis. The protocol can be obtained by contacting the corresponding author.

### 2.1. Eligibility criteria

Studies matching the following criteria were considered for inclusion: English and Japanese peer-reviewed published articles that (1) explored cancer patients’ experiences of financial toxicity related to cancer diagnosis and treatments, (2) were specific to Japan, and (3) focused on experiences of financial toxicity among cancer patients. We selected studies describing patients’ experiences to obtain richer data for an in-depth understanding of the existential burden and suffering. Studies focusing on the patients’ families and healthcare providers were also included.

### 2.2. Study selection

We worked with an expert librarian to develop combinations of search terms (Tables 1, 2; keyword search, e.g., cancer, cost of illness, financial toxicity, Japan) and searched for studies published from inception to August 31, 2022, in the electronic databases PubMed and Igaku Chuo Zasshi (Ichushi); Ichushi is a bibliographic database established in 1903 and updated by the Japan Medical Abstracts Society (JAMAS), a non-profit and non-governmental body. It provides bibliographic citations and abstracts from 2,500 biomedical journals and other serial publications in Japan. PubMed has high coverage of English literature, and Ichushi covers Japanese literature; these two databases can cover the literature on financial toxicity in Japan.

**Table 1:** Search strategy for PubMed.

No	Terms
1	“Neoplasms”[Mesh]
2	Cancer[Title] or neoplasm*[Title] or carcinoma[Title] or tumor*[Title] or tumor*[Title] or oncolog*[Title] or chemotherap*[Title] or antineoplastic[Title] or radiotherap*[Title] or radiation therap*[Title]
3	1 or 2
4	“Cost of Illness”[Mesh]
5	3 and 4
6	“Health Expenditures”[Mesh]
7	Finance* or financial* or economic* or monetary
8	Toxicit* or hardship* or burden* or stress or distress or challenge* or problem* or implication* or personal
9	7 and 8
10	Debt* or bankrupt* or insolven* or expenditure* or “out of pocket” or “material hardship*”
11	Afford* or “pay for” or payment or cost* or money or financ* or loan*
12	Treatment* or therap* or chemotherap* or surger* or care or healthcare or medical
13	11 and 12
14	5 or 6 or 9 or 10 or 13
15	“Japan/epidemiology”[Mesh] or “Japan/ethnology”[Mesh] or “Japan/statistics and numerical data”[Mesh]
16	Japan[Title/Abstract] or Japan*[Affiliation]
17	15 or 16
18	English[Filter] or japanese[Filter]
19	Qualitative or experience* or phenomenolog* or interview* or survey* or questionnaire* or diary or diaries
20	14 and 17 and 18 and 19

**Table 2:** Search strategy for Igaku chuo zasshi.

No	Terms in Japanese	Terms translated in English
1	Keizai dokusei	Financial toxicity
2	Keizai teki sutoresu or Keizai teki konnan	Financial stress or financial burden
3	Gan kanja	Cancer patient
4	1 or 2	1 or 2
5	3 and 4	3 and 4
6	Original paper	Original paper
7	5 and 6	5 and 6

**Figure 1:**
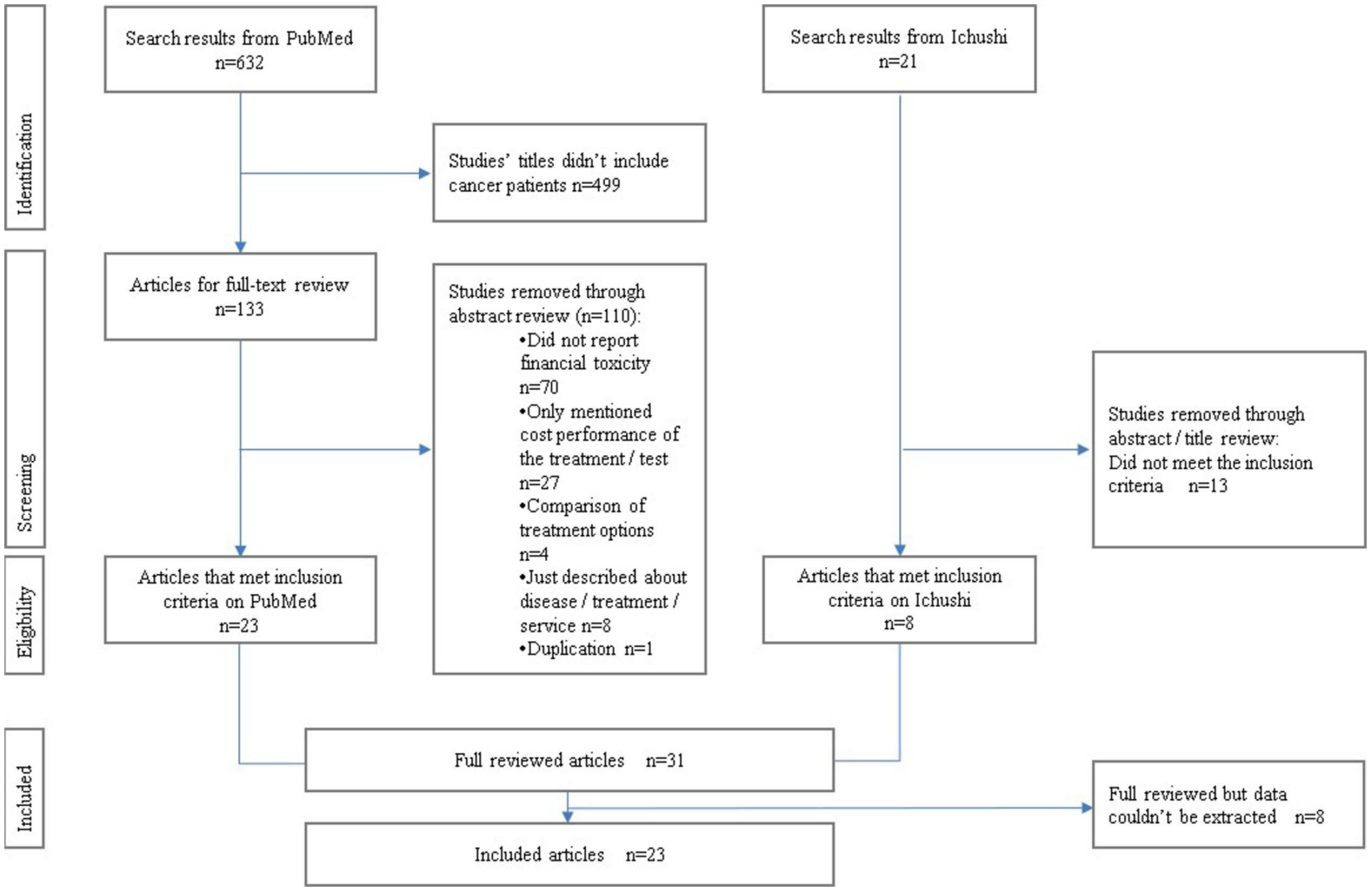
PRISMA-ScR flow diagram.

The titles and abstracts of the eligible studies were screened by two independent reviewers (YI and KO) using the inclusion criteria. The matching rates for the screening were 92.7% (PubMed) and 76.1% (Ichushi). Two independent reviewers (YI and KO) assessed the full texts of the selected studies. Any differences of opinion between reviewers during the search process were resolved through discussion. We excluded (1) studies that did not include patients with cancer, (2) did not reflect patients’ backgrounds, (3) case reports, (4) guidelines, and (5) studies containing inadequate data. The reference lists of all the included studies were screened for additional studies. The results of the search are presented in a PRISMA-ScR flow diagram (Figure 1).

### 2.3. Data extraction and content analysis

As there is no unified or standard definition of financial toxicity, we first extracted different definitions of financial toxicity and the associated risk factors from relevant literature. Furthermore, as there has been no consistent explanation in the past for the categorization of financial toxicity, we used the model literature as a guide to classify financial toxicity in terms of three components; coping with a material burden as a cancer-related (or expected) impact, psychological responses, and behavioral outcomes (Udayakumar et al., 2022). Although behavioral outcomes for healthcare providers were not initially considered, we analyzed the text of the included studies to see if there was an explanation for the impact of patient financial toxicity on behavioral outcomes for healthcare providers. The analyzed data included details about the participants, concepts (i.e., the focus of the study), context (i.e., details about the specific setting), methods, and main findings relevant to the review question.

We created a template for data extraction using Microsoft Excel 2016 software, and two researchers, a general internist and a researcher with a doctoral degree in nursing (YI and KO) independently extracted the relevant text according to the extraction requirements. The extracted data were summarized, and the collated information was reviewed and presented in a tabular form using Microsoft Excel 2016. Then, the extracted main findings were categorized into five themes: (1) Description of financial toxicity, (2) Risk factors for financial toxicity, (3) Coping strategies for material burden, (4) Psychological reactions, and (5) Behavioral outcomes (Table 3). Any differences of opinion between reviewers were resolved through discussion under the guidance of a supervisor (MF).

**Table 3:** Studies reporting patients’ and families’ experience of financial toxicity following cancer diagnosis.

References	Description of financial toxicity	Risk factors for financial toxicity	Three components of financial toxicity		
			Coping strategies for material burden	Psychological reactions	Behavioral outcomes
Aoyama et al. (2021)	Of 491 bereaved family members of cancer patients, 19% reported moderate/severe livelihood concerns. After bereavement, 28% reported worsening of financial status Prevalence of possible complicated grief and major depressive disorder were 9 and 22%, respectively.	Patient’s sex (male), bereaved family member’s sex (female), patient’s age (younger), relation to the patient (spouse), educational background (shorter), current annual income (lower), annual income during caregiving (lower), and whether the family caregiver shared a livelihood with the patient (yes) were significantly associated with. Current annual income, annual income during caregiving, and retirement or leave due to caregiving were significantly related to concerns about current financial status.	Not Applicable	Bereaved family member who reported worsening in financial status after bereavement reported a significantly higher percentage of possible major depressive disorder and complicated grief.	Not Applicable
Honda et al. (2019)	Median COST score was 21. Mean score was 12.1 (SD 8.45; range 0–41).	Older age and higher household savings were significantly associated with higher COST score, which indicates lower FT. Nonregular employment, retirement because of cancer, and use of strategies to cope with the cost of cancer care expenses were significantly associated with lower COST score, which indicates higher FT.	Strategies to cope with the cost of cancer care included using savings to pay for cancer treatment, cutting spending on leisure, and cutting spending on food or clothing.	Not Applicable	Not Applicable
Honda et al. (2019)	Of the 11 patients responded to the questionnaire, the median COST score was 22 (range, 6–29) and mean score was 20.18 (SD, 8.17). Five and 2 patients suffered grade 1 and grade 2 of financial toxicity, respectively.	Not Applicable	36% of patients cut spending on food or clothing, 36% on leisure and 64% used their savings to pay for cancer treatment.	Not Applicable	Not Applicable
Kodama et al. (2012)	Of chronic myelogenous leukemia (CML) patients who were given an imatinib prescription in 2000, 41.2% felt that their medical expenses constituted a heavy financial burden because imatinib was not approved in Japan. This number and ratio rising to 70.8% in 2005, and 75.8% in 2008. Five patients who had no household income spent their savings on medical expenses.	Not Applicable	Not Applicable	Not Applicable	31.7% of CML patients considered imatinib treatment discontinuation because of the financial burden that its use created. Imatinib treatment was temporarily suspended by 2.6% patients because of financial reasons.
Ohno et al. (2020a)	Not Applicable	For annual indirect costs, without adjustment, non-caregivers were estimated to incur significantly less indirect costs than caregivers in both groups. No significant differences in absenteeism cost and indirect cost were observed between caregivers of cancer patients and caregivers of other conditions.	More caregivers of cancer patients had additional cancer insurance (49.8% vs. 30.7% of non-caregivers and 34.2% of other caregivers) and additional severe disease insurance (18.3% vs. 6.5% of non-caregivers and 10.2% of other caregivers) than the other two groups.	A significantly lower proportion of non-caregivers had depression, anxiety, insomnia, headache, migraine, and gastrointestinal problems compared with caregivers in both groups.	Not Applicable
Sasaki et al. (2022)	35% used all or a portion of their savings on treatment, 30% reported reduced spending on clothing, and 24% reported reduced spending on leisure activities such as vacations. Approximately 7.5% of participants reported withdrawal or change of cancer treatment for financial reasons.	Financial difficulties in coping with cancer treatment expenses such as using up all or a portion of one’s savings and subjective financial burden were significantly related to withdrawal or change of cancer treatment (recommended by physicians/based on patient request).	The logistic regression analysis showed that “Cost of care,” “patient had financial concerns,” “patient had cut down on living expenses to pay for cancer treatment,” and “consulted with medical staff about financial issues” had a significant correlation with treatment withdrawn or change (recommended by physicians/based on patient request).	Not Applicable	Approximately 7.5% of participants reported withdrawal or change of cancer treatment for financial reasons.
Takura et al. (2016)	Of 172 experienced Japanese oncologists, 71.3% cited a positive perception (i.e., they felt access to treatment options was adequate) and 28.7% a negative one, regarding the patient’s access to effective medical treatment, irrespective of treatment cost. Regarding the influence of medical expenses on treatment decisions by oncologists, 86% considered potential expenses. Regarding the perceived influence of out-of-pocket expenses on treatment decisions made by patients, 88.4% believed that the patients considered potential expenses before deciding treatment. Of those who answered “with high frequency” to the question about communicating with the patients and their families regarding treatment decisions, 84.6% believed that the patients considered potential expenses before deciding treatment.	Not Applicable	Regarding the influence of medical expenses on treatment decisions by oncologists, 86.0% responded that they considered potential expenses. Further analysis by the area of specialization showed that 93.9% of gastroenterologists considered potential expenses, whereas 36.4% of medical oncologists did not.	Not Applicable	Regarding the maximum allowable public medical expenses to prolong the life expectancy of a cancer patient by 1 year, 41.0% of oncologists believed that the maximum allowable medical expenses for cancer treatment should be≤4million yen/LY, with 39.8% reporting a value of 4–8 million yen/LY
Mitsuki et al. (2010)	In a survey of patients and their families 1 week after discharge after cancer treatment, patients were concerned about their jobs and families, and families were concerned about treatment and financial burdens. The most common concerns were the future course (prognosis) (78.4% for patients and 87.3% for families), followed by symptoms (24.3 and 33.3%), financial burden (20.3 and 23.0%), work (20.3 and 11.9%), treatment (19.6 and 29.4%), family (18.9 and 4.7%), death (12.8 and 14.3%), taking care of oneself (10.8 and 5.6%), and relationship with doctor (7.4 and 8.7%).	Not Applicable	In the “What the patients and their families want to know now,” 50.7% of the patients and 49.2% of the family members answered about recurrence/metastasis, 29.7 and 27.0% about diet, 25.7 and 34.9% about side effects and managements, 25.7 and 26.2% about anticancer treatment, 23.6 and 317% about the course of the disease, 18.2 and 15.9% about exercise, 14.9 and 15.1% about elimination, 14.3 and 20.6% about the effects and complications of the treatments, 13.5 and 4.0% about sleep, 10.1 and 11.9% about available services and social systems, and 10.1 and 12.7% about pain control. Regarding the “desired support” of the patients and their families, 29.7% of the patients wanted to discuss treatment, 9.5% financial matters, and 5.4% living matters, while 24.6% of the families wanted to discuss treatment, 11.1% living matters, and 7.1% financial matters.	Not Applicable	Not Applicable
Komura et al. (2011)	Regarding requests for improvement of cancer treatment and palliative care, the most common need of the patient survey was for improvement of the health care system (50.2%), followed by staffing (30.3%) and treatment (25.8%). 10.3% of patients and 8.0% of bereavement families wanted a reduction in the financial burden, 8.1% of patients and wanted improvements in the hospital system such as shorter waiting times for outpatient visits, development of outpatient nursing care, collaboration with other departments, and after-hours hospital care.	Not Applicable	Not Applicable	Not Applicable	Not Applicable
Tsuchiya et al. (2018)	Not Applicable	Not Applicable	Among outpatients who were working at the time of cancer diagnosis, about 90% recognized the high-cost medical expense benefit, while about 40% for the sickness and injury benefit and 70% for the medical expense deduction. Information was obtained either by the patients or by their family members, and information provided by healthcare providers was relatively low. Patients who continued to work obtained information from their employers. About 70% of the patients used the high-cost medical expense, 20% used the sickness benefit, and 40% used the medical expense deduction.	Not Applicable	Not Applicable
Okubo (2013)	The spouses of cancer patients felt difficulties in dealing with the financial burden of cancer treatment, such as the high medical costs associated with cancer. Financial problems weigh heavily on the spouse of the cancer patient due to changes in lifestyle patterns. Spouses struggled with the costs associated with cancer treatment itself, such as treatment and hospitalization costs, as well as having to deal with reduced income or loss of income due to spousal leaves of absence or retirement.	Not Applicable	Not Applicable	Not Applicable	Not Applicable
Osono et al. (2012)	The financial toxicity of cancer patients as perceived by home health nurses caring for terminally ill patients was the increased payment burden on cancer patients when public long-term care insurance was not available. The nurses were conflicted about the increased burden on the patient while benefiting the home care facility.	Not Applicable	As a solution to the financial burden of cancer patients, the home health nurses mentioned that they and the patients/families should evaluate the services and their compensation, consider the balance with the financial burden, and think together if there is any way to reduce the financial burden.	Not Applicable	Not Applicable
Oizumi et al. (2018)	In a questionnaire survey of family members of pancreatic cancer patients undergoing treatment, the families’ perceived financial burden caused by treatment was very burdensome (6%) and somewhat burdensome (37.3%). Regarding the financial burden of treatment and care, families who reported having a burden had significantly lower “vitality,” “social life function,” and “mental health” as assessed by the SF-12 quality-of-life scale compared to families who did not have a burden.	Not Applicable	Not Applicable	Not Applicable	Not Applicable
Sugiyama et al. (2017)	Perceived financial burden of caregivers of cancer patients was associated with younger age, lower household income, separation from spouse, change in employment (reduced working hours, resignation), reduced income, presence of family members requiring care, less favorable relationship with the patient, and younger patient.	Factors that increased the family caregiver’s financial burden were caregiver’s younger age (*p* < 0.01), lower household income (*p* < 0.01), separation from spouse (*p* = 0.03), reduced working hours (*p* = 0.02), resignation (*p* = 0.04), decreased income since the patient diagnosis (*p* < 0.01), having another family member in need of care (*p* < 0.01), not having a good relationship with the patient (*p* < 0.01), having other family members in need of care (*p* = 0.03), did not feel they had a good relationship with the patient (*p* < 0.01), and the patient was younger (*p* < 0.01).	Regarding the relationship between family members and cancer patients, a good relationship with the patient enhanced the family caregivers’ positive perception of caregiving, and lowered the life obstacles and financial burdens caused by caregiving.	Factors that increased the psychological burden of family caregivers were that the caregiver was female (*p* = 0.02), unmarried (*p* < 0.01), income decreased after the patient diagnosis (*p* = 0.03), presence of another family member requiring care (*p* < 0.01), patient was under 60 (*p* = 0.04), and the patient was in need of care (*p* = 0.02).	Not Applicable
Hayashida et al. (2005)	Cancer patients receiving outpatient treatment experienced financial hardship caused by their treatment, which was categorized into the following three groups: “significant burden of treatment costs,” “high cost of drugs,” and “pressure on family finances from treatment costs.” Specifically, the cancer patients expressed that “if I start using drugs that are not covered by insurance, I have to pay however much money I have to pay,” “100,000 yen a month, three times a month,” “It has become more difficult financially,” and “It’s hard on my family financially.	Not Applicable	Cancer patients mentioned that they did not address the financial burden of their treatment.	Not Applicable	Not Applicable
Taguchi et al. (2019)	Among breast cancer patients who were working, 19% had their employment changed (insurance changed) at least once before or after surgery (once was 69% and twice or more was 31%). The average time between surgery and changing jobs (insurance) was 8.7 months. Of those who changed jobs, 10% changed jobs by the end of the month prior to surgery. Forty-two percent had changed jobs by the fourth month, and 65% had changed jobs by the twelfth month after surgery.	Treatment had a significant effect on job change, with those who underwent mastectomy more likely to change jobs than those who underwent breast-conserving surgery, and patients on multiple-drug therapy more likely to change jobs than those who did not. On the other hand, there was no statistically significant relationship between age, type of insurance, and job change.	Not Applicable	Not Applicable	Not Applicable
Umezawa et al. (2015)	Not Applicable	Lower income was associated with unmet medical-psychological, financial, and social-spiritual needs.	Patients diagnosed with cancer within 10 years reported that support for their unmet needs from medical professionals was preferred for most of the needs except for financial needs. Non-medical professionals (e.g., social welfare, labor union, job-coordination center, professional helpers, and insurance company) were the preferred source of support for financial needs.	Not Applicable	Not Applicable
Ito et al. (2015)	A high proportion of newly diagnosed cancer patients (75.8%) had returned to work. Non-regularly employed survivors were less likely to return to work (odds ratio = 5.03; 95% confidence interval, 1.18–21.35). Individuals with poor health, advanced-stage tumors, of advanced age, and women were significantly less likely to return to work. Only 52.8% of non-regular employees continued to be employed, and their income decreased by as much as 61.1%.	Non-regularly employed survivors were more likely not to return to work, followed by poor health status, being female, having an advanced-stage tumor, and being advanced in age. Compared with 80.5% of the self-employed workers, 79.2% of the regular employees in the public sector and 69.3% of the regular employees in the private sector, only 52.8% of the non-regular employees continued in the jobs they held at the time of diagnosis. At diagnosis, 25.1% of the regularly employed patients had an annual income <2 million yen. This percentage increased to 43.2% at ≥1 year after diagnosis. For self-employed workers, the change was from 18.9 to 42.6%, and for regular employees, the change was from 18.8 to 41.1%. For the non-regular workers, 73.5% earned <2 million yen annually at the time of diagnosis, and this percentage was unchanged at ≥1 year after diagnosis. However, 61.1% of non-regular workers earned a lower income at ≥1 year after diagnosis.	Not Applicable	Not Applicable	Not Applicable
Munakata et al. (2022)	Chronic myelogenous leukemia (CML) physicians did not recommend an optimal regimen to 6.53% of their patients per year because of cost. Some patients refused, discontinued, reduced, or skipped treatment owing to cost. In total 10–20% of patients with CML may receive non-optimal treatment owing to treatment cost. Among CML physicians versus transplant-ineligible MM physicians, 59.0 and 54.7%, respectively, said that they consider the balance between drug cost and efficacy when choosing a regimen for their patients, including newly diagnosed cases, and 46.7 and 53.8% said that they consider the balance between drug cost and efficacy when choosing a regimen for later lines of treatment. On the other hand, only 21.9 and 20.8% said that they proactively reduce dose or skip treatment to reduce the treatment cost burden on patients.	Not Applicable	Not Applicable	Not Applicable	While treatment cost was not an issue for most patients, CML physicians did not recommend an optimal regimen to 6.53% of their patients per year because of cost. Moreover, 1.51% of these patients refused treatment owing to cost and, among patients who began treatment, 1.97% discontinued, 4.17% reduced their dose, and 3.48% skipped a dose owing to cost. This suggests that 10–20% of patients with CML overall may receive non-optimal treatment owing to treatment cost
Watanabe et al. (2021)	Regarding the first-year costs per patient for the five common cancers, lung cancer obtained the highest median overall costs per patient (2,508,789 JPY), while breast cancer showed the lowest (1,559,274 JPY). According to clinical stage, stage III cancers exhibited the highest median inpatient costs, except for colorectal cancer for which the highest inpatient cost was for stage IV.	Not Applicable	Not Applicable	Not Applicable	Not Applicable
Saito et al. (2014)	Among 105 women diagnosed with breast cancer, 29.5% lost their jobs, and 12 could not find another job after cancer diagnosis. Nearly half of the respondents (47.6%) reported a decrease in personal income after diagnosis. Multiple logistic regression analysis revealed that non-regular or part time workers were significantly more likely to lose their jobs compared with regular, full-time workers.	Not Applicable	Not Applicable	Not Applicable	Not Applicable
Kaneko et al. (2020)	According to data from a nationwide population-based longitudinal survey, male workers are more likely to quit their job in the year they are diagnosed with cancer, and also in the following year compared to the matched control. Contrastingly, female workers are more likely to quit their job immediately after being diagnosed with cancer; however, this effect totally disappears when considering likelihoods for the following year. Cognitive workers are more prone to quit their job in the year of diagnosis by 11.6 percentage points, and this effect remains significant, 3.8 percentage points, in the following year. On the other hand, for manual workers the effect during the year of diagnosis is huge. It amounts to 18.7 percentage points; however, the effect almost disappears in the following year.	Not Applicable	Not Applicable	Not Applicable	Not Applicable
Okada et al. (2015)	The most common reason for continuing to work was “for a living” (50%). Other reasons were “working was natural for me,” “to earn money for child’s care and medical costs,” and “too difficult to find reemployment.” Of the 10 mothers who quit, 5 mothers reported financial problems.	Not Applicable	More than 80% of mothers who continued to work reported that “support from employers for work and family life” (90.0%) and “improvement of extended leave systems for health problems of family members and making the office atmosphere more amenable for extended leave” (80.0%) were needed. Of the 32 mothers who worked at the time of diagnosis, 8 continued working without change, 1 changed working hours, and 1 decreased working days. Of the 10 mothers who quit, 5 mothers reported financial problems.	Not Applicable	Not Applicable

**Figure 2:**
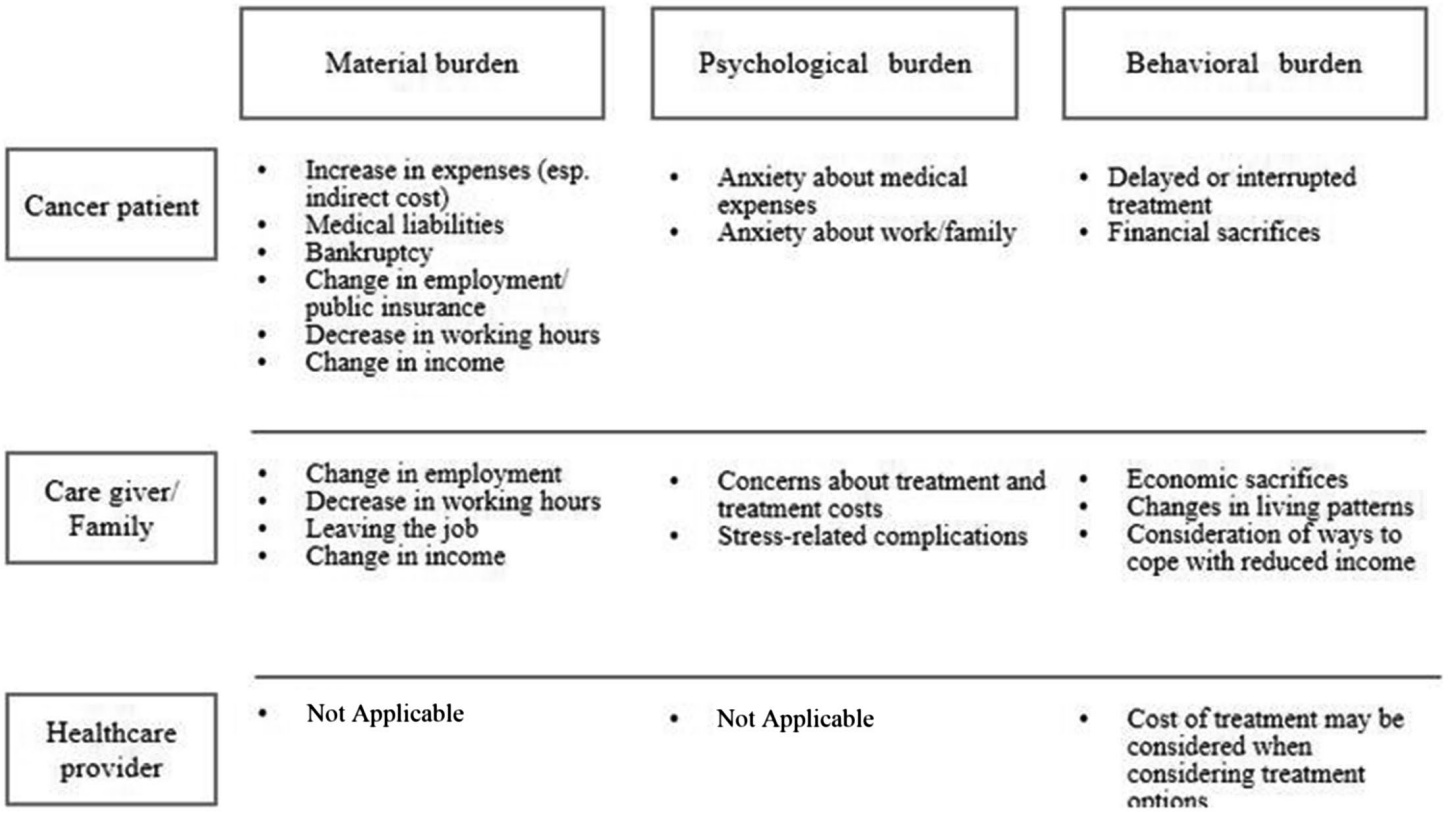
Differences in financial toxicity caused by stakeholders.

These five themes were referenced from the previous study in low- and middle-income countries (Udayakumar et al., 2022), which was in line with our aim to achieve an overview of financial toxicity. We summarized the extracted data in Table 3 according to each theme. We reorganized the overview of financial toxicity in Table 3 into financial, psychological, and behavioral toxicity in Figure 2, respectively, for patients, families, and healthcare providers. We found an additional theme (healthcare providers’ behavioral hardships) and added it as a sixth theme.

## 3. Results

### 3.1. Search results and characteristics

We searched 632 citations from PubMed and 21 from Ichushi and excluded duplicates. Based on the titles and abstracts, 499 articles were excluded, and 154 full-text articles were evaluated for eligibility. Of these, 123 were excluded for the following reasons: 70 did not report financial toxicity; 27 only mentioned the cost performance of the treatment (the main theme was a comparison of treatment options); and 8 only described disease/treatment services. As two reports from one survey were included, a follow-up version with a more detailed description was adopted. Thirty-one articles were selected for a full-text review (Figure 1).

Eight studies mentioned risk factors for financial toxicity. Twenty-three studies reported descriptions of financial toxicities (twenty quantitative and three qualitative studies). Three studies examined psychological reactions. Twelve studies reported coping strategies for financial toxicity. Four studies investigated the impact on treatment outcomes.

### 3.2. Literature overview of financial toxicity experienced by patients with cancer and their families in Japan

Newer chemotherapeutic drugs are generally expensive, and as prognosis improves, the duration of treatment is lengthened, increasing the financial burden of treatment. The financial burden of cancer treatment that affects individuals’ lifestyles has been defined as “financial toxicity,” which should be treated in the same way as physical treatment-related toxicity. In Japan, approximately 70% of patients (Honda et al., 2019) and 30% of their families (Aoyama et al., 2021) experience financial toxicity. Moreover, 30% of healthcare providers consider that medical costs prevent patients from accessing appropriate treatment (Takura et al., 2016). Stomach, lung, colorectal, liver, and breast cancers are the five major types of cancer in Japan. The median total cost per patient was highest for lung cancer (2,508,789 JPY) and lowest for breast cancer (1,559,274 JPY) (Watanabe et al., 2021). Kodama et al. and Munakata et al. reported the financial burden on patients with chronic myelogenous leukemia and their physicians’ perceptions of treatment selection (Kodama et al., 2012; Munakata et al., 2022). With respect to age groups, patients in their 60s and 70s had the highest cancer treatment costs for all cancer types, except breast cancer. More than 50% of breast cancer treatment costs were incurred by patients aged <60 (Watanabe et al., 2021).

Families of cancer patients experienced difficulties dealing with the financial burden of high medical costs and reduced income due to changes in lifestyle patterns (Okubo, 2013). In families of patients with pancreatic cancer undergoing treatment, 37.3 and 6% felt financial burden as “somewhat burdensome” and “very burdensome,” respectively (Oizumi et al., 2018). Previous studies have reported that during treatment, patients are concerned about cancer prognosis (78.4%), symptoms (24.3%), and finance and work (20.3 and 20.3%, respectively), while family members are concerned about treatment (29.3%) and financial burden (23.0%), followed by cancer prognosis (87.3%) and symptoms (33.3%) (Mitsuki et al., 2010). Regarding palliative care, 10.3% of patients and 8.0% of bereaved families wanted a reduction in the financial burden (Komura et al., 2011). Oizumi et al. reported significant relationships between lower levels of “vitality,” “social functioning,” and “mental health” as assessed using the quality of life scale and the financial burden of care and treatment (Oizumi et al., 2018).

### 3.3. Six main themes of financial toxicity

**Figure 3:**
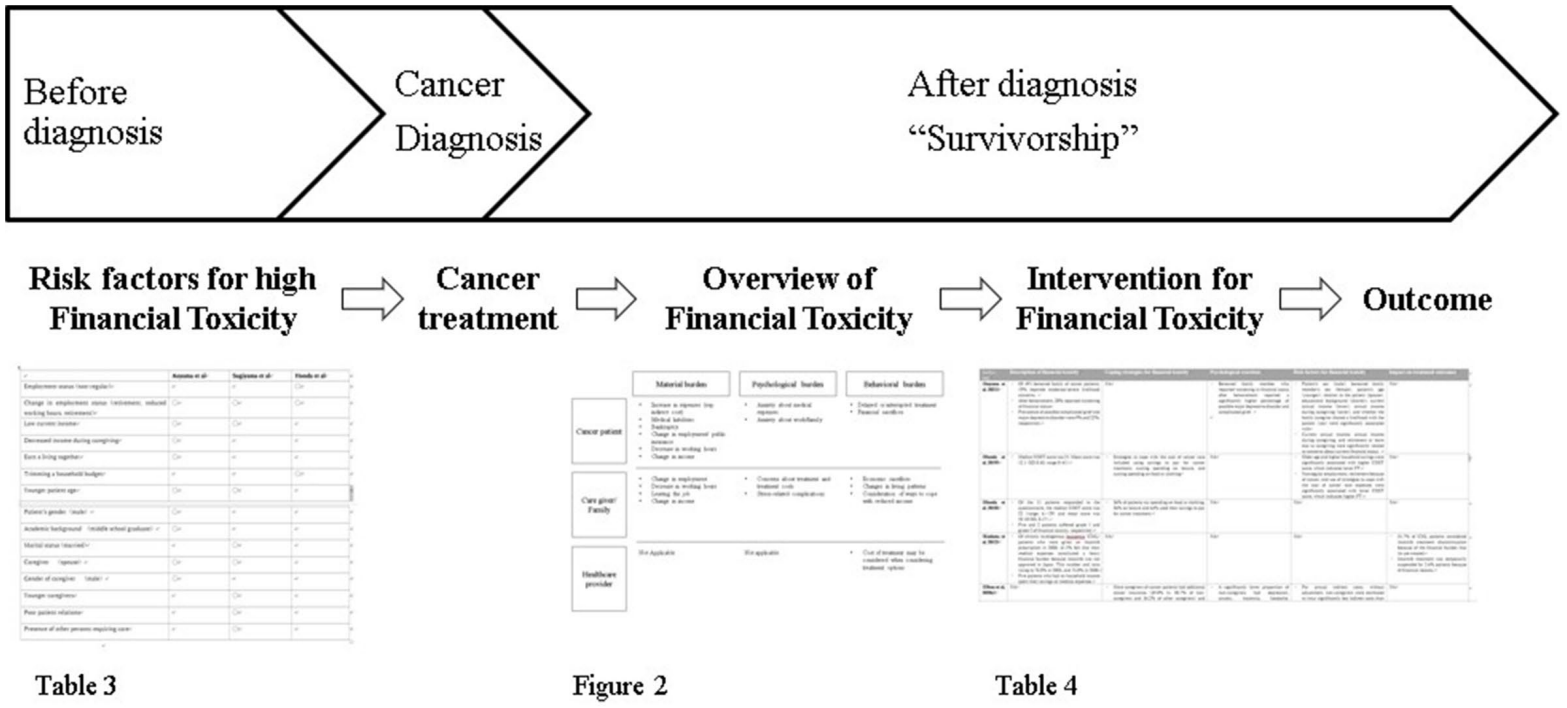
Overview of figures and tables.

We originally summarized results along five themes but because of the new recognition of behavioral toxicity as a characteristic of healthcare providers in Japan, we report on six themes. Figure 3 shows a chronological overview of results during cancer survivorship.

#### 3.3.1. Description of financial toxicity: patients and their families encountered material, psychological, and behavioral burden

The various hardships experienced by the patients and their families are shown in Figure 2. Financial toxicity includes not only the material burden but also the extent to which such financial burden affects patients’ subjective well-being (Honda et al., 2018). Financial toxicity among patients receiving outpatient treatment includes the significant treatment cost, high cost of drugs, and the pressure of treatment costs on household finances (Hayashida et al., 2005). Hayashida et al. reported that patients who participated in their study did not address the material burden of their treatment (Hayashida et al., 2005).

Cancer patients experience material (e.g., increase in expenses, medical liabilities, bankruptcy, change in employment public insurance, decrease in working hours, and change in income), psychological (e.g., anxiety about medical expenses, work, and family), and behavioral burdens (e.g., delayed or interrupted treatment and financial sacrifices) (Figure 2). Among 105 women diagnosed with breast cancer, 31 (29.5%) lost their jobs and 50 (47.6%) reported decreased personal income after diagnosis (Saito et al., 2014). Saito et al. also reported that non-regular or part-time workers were significantly more at risk of losing their jobs than regular or full-time workers (Saito et al., 2014). Ito et al. reported a high return-to-work immediately after diagnosis rate of 75.8% for cancer survivors, and a lower rate for those in informal employment (Ito et al., 2015). In addition, female patients and patients with poor health, advanced cancer, and older age had significantly lower return-to-work rates. The rate of continued employment among informal workers remained at 52.8%, and their income decreased to 61.1% (Ito et al., 2015). According to data from a nationwide population-based longitudinal survey (Kaneko et al., 2020), male and white-collar workers were more likely than matched controls to quit their jobs in the year of diagnosis and the following year. Whereas, female workers had a higher risk of quitting their jobs immediately after cancer diagnosis; however, this effect completely disappeared when considering the possibility in the following year. For manual workers, although the effect was quite large in the year they were diagnosed, it was almost eliminated in the following year (Kaneko et al., 2020).

Patients’ family members also bear financial (change in employment, decrease in working hours, leaving the job, and change in income), psychological (concerns about treatment and costs, and stress-related complications), and behavioral burdens (financial sacrifices, changes in living patterns, and consideration of ways to cope with reduced income). Regarding the impact of childhood cancer, Okada et al. reported financial problems for mothers who were working when their children were diagnosed (Okada et al., 2015). Eight of the 32 mothers continued to work as before, one changed her working hours, and one reduced the number of working days. The most common reason for working was to make a living (50%). Other reasons included working normally, earning money for childcare and medical expenses, and difficulty in finding new employment. Of the 10 who quit their jobs, five mothers expressed economic problems (Okada et al., 2015).

#### 3.3.2. Description of financial toxicity: there would be a behavioral impact on healthcare providers who faced patients’ financial burden

The model of previous studies especially in the United States did not include healthcare providers’ perspective, but literature in Japan suggested that decision making for treatment choice is influenced by patients’ financial situation. They experience behavioral burdens, considering the cost of treatment options for patients with cancer. These components are shown in Figure 2. Munakata et al. and Takura et al. examined physicians’ perceptions of optimal treatment selection and cost burden for their patients (Takura et al., 2016; Munakata et al., 2022). Of the 172 experienced Japanese oncologists, 86% considered potential medical expenses when deciding treatment options, and 88% believed that the patients considered potential expenses when deciding their treatment (Takura et al., 2016). While the cost of treatment was not a major problem for most patients with chronic myelogenous leukemia, physicians did not recommend an optimal regimen to 6.5% of their patients per year because of the cost. Moreover, 1.5% of these patients refused treatment owing to cost, and among patients who started treatment, 2.0% discontinued, 4.2% reduced their dose, and 3.5% skipped the dose owing to cost. This suggests that 10–20% of patients with chronic myelogenous leukemia may receive non-optimal treatment due to treatment costs. Approximately 50% of the physicians considered the balance between drug cost and efficacy when selecting a patient’s regimen, including newly diagnosed cases and later lines of treatment. However, only 20% reported proactively reducing the dose or skipping treatment to reduce the treatment cost burden on the patients (Munakata et al., 2022) (Figure 2).

On the other hand, the literature did not reveal material or psychological burdens for healthcare providers. The concept of financial toxicity itself is subjective, and there is no objective method to evaluate financial toxicity. Comprehensive Score for Financial Toxicity (COST) is globally used for evaluating the level of financial toxicity. COST score can be useful to quantitatively evaluate patients’ financial toxicity, which has been assessed sensitively. Since financial toxicity can occur at any time after diagnosis, it would be useful to screen all patients from the time of diagnosis and share the risk with healthcare providers. Future studies will require the development of a scale that can be used to quantitatively assess, track, and compare financial toxicity, not only among cancer patients but also among their caregivers and healthcare providers.

#### 3.3.3. Risk factors for financial toxicity: patient characteristics are related to a higher level of financial toxicity

Of eight studies, only three studies reported high-risk factors for financial toxicity; specifically, changes in employment, decreased hours of work, job turnover, and income changes occur among families (Sugiyama et al., 2017). In addition to direct financial problems such as high medical, treatment, and hospitalization costs for cancer treatment, they struggled to cope with reduced income, loss of income due to spousal leave, or retirement due to changes in lifestyle patterns (Okubo, 2013). When long-term care insurance is unavailable, 30% of the medical expenses are borne, and the use of nursing care stations and other services leads to increased uncovered expenses for family members. The financial burden faced by patients and the provision of care are discordant (Osono et al., 2012), which is problematic.

#### 3.3.4. Psychological reactions: various psychological responses are observed among cancer patients and families

Psychological burden increased based on the following factors: female family members, unmarried patients, decreased income since the patient became ill, other family members needing care, younger patient age, and high need for care (Sugiyama et al., 2017; Aoyama et al., 2021). In addition, those with a sense of financial burden reported higher rates of major depressive disorder and increased stress-related complications for family members (Aoyama et al., 2021), as did those whose financial situations had changed since the patient’s death (Ohno et al., 2020a).

#### 3.3.5. Coping strategies for financial toxicities: multiple coping behaviors are observed with the help of various stakeholders

Coping with financial toxicity includes trimming household finances (Honda et al., 2019), using savings to pay for cancer treatment (Honda et al., 2018, 2019; Sasaki et al., 2022), consulting with healthcare providers (Sasaki et al., 2022), deepening the understanding of public financial support systems and medical service contents, such as sickness benefits and medical expense deduction (Komura et al., 2011; Osono et al., 2012; Tsuchiya et al., 2018), building good relationships between patients and families (Sugiyama et al., 2017), and obtaining appropriate insurance (Takura et al., 2016; Ohno et al., 2020b; Sasaki et al., 2022). Among outpatients who were working when they were diagnosed with cancer, around 70% used high-cost medical expenses, 20% used the sickness benefit, and 40% used the medical expense deduction; information obtained either by the patients or by their family members, or given by healthcare providers was relatively low (Tsuchiya et al., 2018). Patients who continued to work obtained information and support from their employers (Okada et al., 2015; Tsuchiya et al., 2018). Regarding the relationship between family members and cancer patients, Sugiyama et al. reported that good relationships lowered the financial burden caused by caregiving (Sugiyama et al., 2017).

Umezawa et al. reported that cancer patients diagnosed within 10 years preferred support from healthcare providers for most unmet needs, including medical, psychological, social, and spiritual, with the exception of financial needs (Umezawa et al., 2015). However, non-medical professionals (e.g., social welfare, labor unions, job coordination centers, professional helpers, and insurance companies) are a favorable source of support for financial needs (Umezawa et al., 2015). Osono et al. reported that home health nurses state that healthcare providers, patients, and families should evaluate the service and their compensation to consider the balance between service use and financial burden and think of strategies to reduce financial toxicity. Hayashida et al. reported that cancer patients did not address the financial burden of their treatment (Saito et al., 2014).

#### 3.3.6. Impact on treatment outcomes: financial toxicities, especially treatment cost, can affect treatment outcomes

Non-optimal treatment due to treatment costs occurs in Japan for patients with chronic myelogenous leukemia and multiple myeloma who cannot be offered a transplant. However, this may be limited to a small percentage of patients. While treatment cost was not a major problem for most patients, physicians treating chronic myelogenous leukemia did not recommend an optimal regimen for 6.5% of their patients per year due to the cost. Moreover, 1.5% of these patients refused treatment owing to cost, and among patients who began treatment, 2.0% discontinued, 4.2% reduced their dose, and 3.5% skipped the dose owing to the cost. This indicates that 10–20% of patients with chronic myelogenous leukemia may receive non-optimal treatment due to treatment costs (Munakata et al., 2022).

A significant correlation between anxiety and treatment interruption or change was found in patients with financial difficulties and anxiety (Sasaki et al., 2022); more than 30% of patients considered treatment interruption for financial reasons (Kodama et al., 2012), and approximately 7.5% of patients reported withdrawal or change of cancer treatment for financial reasons (Sasaki et al., 2022). Approximately 40% of patients answered that the maximum allowable cancer treatment cost is less than 4 million yen/year and 40% answered 4–8 million yen/year (Takura et al., 2016).

Regarding the impact of medical costs on physicians’ treatment decisions, 86.0% of the physicians considered potential medical costs. Regarding the impact of co-payments on patients’ treatment decisions, 88.4% of the patients indicated that they would consider co-payments before deciding on a treatment plan (Takura et al., 2016).

### 3.4. Level of financial toxicity among Japanese patients indicated by the comprehensive score for financial toxicity

The Comprehensive Score for Financial Toxicity (COST) is the most widely used and highly reliable scale for measuring financial toxicity in cancer patients (Zhu et al., 2022). We found several articles that adopted the COST for Japanese patients. The scale consists of 11 items with 0–4 response values and a total score ranging from 0 to 44, with a lower score representing a greater degree of financial toxicity. The COST is intended to holistically capture financial toxicity, including psychological distress and physical effects. A score of 26 or above is treated as grade 0, 14–25 as grade 1, 1–13 as grade 2, and 0 as grade 3; grades 1 and above (score of 25 or below) were evaluated as having financial toxicity (de Souza et al., 2017). Huntington et al. reported that in the US, median COST scores were 23 (Huntington et al., 2015). Only three studies, published by the same author, mentioned the COST score of Japanese cancer patients, showing a median score of 21 (Honda et al., 2019) and 22 (Honda et al., 2018); furthermore, 63% had Grade 1–2 financial toxicity among patients undergoing chemotherapy (Honda, 2018).

## 4. Discussion

We conducted a scoping review of the literature focusing on qualitative experiences of financial toxicities experienced by cancer patients in Japan. The study revealed that even in Japan, where universal health insurance is available, patients with cancer experience the same level of financial toxicity as in other countries, which is in line with the findings of previous studies on publicly funded healthcare countries (Longo et al., 2020). The primary strength of our study is its comprehensive compilation of the Japanese and English literature on financial toxicity in Japan and its comparison with previous studies in low- and middle-income countries (Udayakumar et al., 2022) and high-income countries other than Japan (Smith et al., 2019; Longo et al., 2020; Hussaini et al., 2022). While evidence-building on cancer survivorship in Japan is still insufficient, this study is significant because the results were extracted from both Japanese and English articles. The credibility of the evidence coverage is high because it was performed according to the review protocol (PRISMA).

Throughout the review, we organized the process of financial toxicity (Figure 3), which usually occurs between the time of cancer diagnosis and death. The high-risk group may have originally experienced financial toxicity (Table 4). Multiple studies cited low income, younger patient age, and caregiver (spouse) status as risk factors. Being female, being unmarried, and caring for family members were associated with psychological aspects of financial toxicity. Financial toxicity may also contribute to increased depression and stress-related complications in the patient’s family. These are consistent with some of the factors reported in a systematic review of risk factors (Smith et al., 2019).

**Table 4:** High-risk factors for high economic toxicity included in previous Japanese studies (all are observational studies).

	Aoyama et al (Reference No.12)	Sugiyama et al (Reference No. 34)	Honda et al (Reference No.11)
Employment status (non-regular)			〇
Low current income	〇	〇	
Decreased income during caregiving	〇		
Earn a living together	〇		
Trimming a household budget			〇
Younger patient age	〇	〇	
Patient‘s gender (male)	〇		
Academic background (middle school graduate)	〇		
Marital status (married)		〇	
Caregiver (spouse)	〇	〇	
Gender of caregiver (male)	〇		
Younger caregivers		〇	
Poor patient relations		〇	
Presence of other persons requiring care		〇	

There are coping strategies on the part of patients and families and support systems from the government and healthcare providers that help deal with financial toxicity. However, the use of the support system for financial toxicity remains limited in Japan (Tsuchiya et al., 2018). Information on these systems was collected mainly by the patients themselves or their family members, and information given by healthcare providers, excluding social workers, was relatively low on the list. Our study clarified that various administrative services are available to patients and their families in Japan; however, poor access to such services may be a factor limiting their use. To overcome these obstacles, some solutions would be to actively encourage patients to ask healthcare providers questions, improve efforts at the field level to link patients to the system, ensure that the government as policymakers review the design of the support system, and improve the method of publicizing the system. More than 80% of physicians and patients consider treatment costs when selecting a treatment. This reveals that the cost of treatment potentially influences decision-making, even in Japan, where the universal health insurance system provides citizens with relatively good access to medical care. Further research is needed to ascertain how proactive interventions by healthcare providers contribute to reducing financial toxicity. In particular, it would be helpful to evaluate the efficacy of tools (e.g., question prompt lists) that encourage patients to proactively discuss financial toxicity with their healthcare providers (Brandes et al., 2015). Hospital-based healthcare providers need to enhance collaboration with social workers and other multidisciplinary professionals to solve patients’ financial toxicity in cooperation with their communities. According to the Basic Plan for Cancer Control issued by the Japanese Ministry of Health, Labor, and Welfare, the functions of patient consultation services need to be strengthened. The Cancer Consultation Support Center, where nurses and medical social workers provide consultation on cancer treatment and survivorship life issues, is accessible to both hospital patients and patients in and out of the community. However, a survey of the cancer patient experience in Japan reveals that the utilization rate of cancer counseling and support centers is low.

Intervention studies on financial toxicity are currently being conducted in the US. The CAFÉ study will provide essential early trial evidence on the impact of financial navigation to reduce cancer-related financial toxicity (Henrikson et al., 2022). Cancer patients in Japan also experience financial toxicity, the same as other publicly funded healthcare countries. It is necessary to effectively connect patients to services and systems that support them and develop policies that are easy to use.

Our scoping review has certain limitations. As there were few studies and reports on financial toxicity in Japan, little information was obtained. Financial toxicity and employment are closely related; however, it was impossible to investigate the reasons that made it difficult to find employment or work in detail. We could not find a nationwide survey in Japan that distinguished the onset of financial toxicity by cancer season, from cancer diagnosis to post-treatment. Patients are difficult to follow after treatment has been completed because they do not appear on insurance reimbursement data. Thus, it remains unclear when cancer patients are most likely to experience financial toxicity after diagnosis. Further research is needed to clarify when financial toxicity increases to provide effective interventions. As we restricted our inclusion criteria to English or Japanese peer-reviewed articles available in PubMed and Ichushi, we may have missed relevant data from non-English or non-Japanese articles or other databases. We did not conduct a quality appraisal of the studies; therefore, we cannot be sure of the quality of the included studies, although this is optional in scoping review (Peters et al., 2015). Most qualitative data included in this review were gathered through interviews or questionnaires, which may have had recall, selection, and/or sampling biases.

## 5. Conclusion

In conclusion, through a scoping review of cancer treatment-related financial toxicity in Japan, we found that patients experienced negative consequences owing to their financial burdens, such as increased cost of treatment, poor adherence to treatment, and anxiety. Considering the factors that affect the feasibility of strategies to address financial toxicities in Japan, the implementation of evidence-based solutions is required to reduce the negative influence of these toxicities among cancer patients. We recommended that the Japanese government should take the initiative to foster evidence making for financial toxicity. Solid evidence will enhance healthcare providers’ recognition. Similarly, the government should inaugurate research groups or formulate a guideline for financial toxicity, which will also be helpful in daily clinical settings.

## Author contributions

The conception and design of this scoping review was done by MF, YI, and KO. The process of article collection and review was conducted by YI and KO. YI, KO and MF contributed to the data analysis and integration. The first draft of the manuscript was written by YI, KO and MF. JS and YU commented on the previous draft. All authors have approved the manuscript as submitted and agree to accept responsibility for any part of the manuscript.

## Funding

This study was funded by the National Cancer Center Research and Development Fund (2022-A-22). The funder has no role and no involvement in the design, conduct, analysis, interpretation of data, or decision to submit the results of this study.

## Conflict of interest

The authors declare that the research was conducted in the absence of any commercial or financial relationships that could be construed as a potential conflict of interest.

## Publisher’s note

All claims expressed in this article are solely those of the authors and do not necessarily represent those of their affiliated organizations, or those of the publisher, the editors and the reviewers. Any product that may be evaluated in this article, or claim that may be made by its manufacturer, is not guaranteed or endorsed by the publisher.
