# Supplementary material for: Cancer treatment-related financial toxicity in Japan: a scoping review
Source: Front Psychol. 2023 Aug 1;14:1205016. doi: 10.3389/fpsyg.2023.1205016 (PMC10428644; doi:10.3389/fpsyg.2023.1205016)
Supplement: Supplementary file 1 [file Table_1.DOCX]

# Supplementary table 1. Study description

| Author, year | Study purpose | Study participants | Study method | Main findings |
| --- | --- | --- | --- | --- |
| (Munakata et al., 2022) | To ascertain the number of patients with chronic myelogenous leukemia (CML) and the transplant-ineligible patients with multiple myeloma (MM) who were not recommended by their physicians for optimal drug treatment or who refused, discontinued, reduced, or skipped treatment owing to the cost. | The survey included 212 hematologists (approximately 8% of all hematologists practicing in Japan) in 212 unique facilities, including 40% (n=140) of all cancer-based hospitals and treatment centers. | Prospective, cross-sectional survey | While treatment cost was not an issue for most patients, CML physicians did not recommend an optimal regimen for 6.53% of their patients per year because of the cost. Moreover, 1.51% of the patients refused treatment owing to the cost. Among patients who began the treatment, 1.97% discontinued, 4.17% reduced their dose, and 3.48% skipped a dose due to the cost. Owing to the treatment cost, 10–20% of patients with CML and 2–4% of the transplant-ineligible patients with MM receive non-optimal treatment. |
| (Sasaki et al., 2022) | To examine the characteristics and impact of the financial burden on cancer treatment from diagnosis to end-of-life, as well as its effect on treatment withdrawal or change of cancer treatment in Japan. | A total of 510 (60%) questionnaires were returned from family members of deceased cancer patients; of those, 59% were male; 56% were 75 years old or older, 72% were unemployed, 16% had financial concerns, 33% had private cancer insurance, and 67% used the high-cost medical care benefit system. | Secondary analysis of the cross-sectional surveys, part of a nationwide survey of bereaved family members of cancer patients in Japan (J-HOPE2016 study) | Of the deceased cancer patients, 7.5% of family participants reported withdrawal from or change in cancer treatment for financial reasons. Financial difficulties in coping with cancer treatment expenses, such as spending all or a portion of one’s savings (OR = 2.14, 95% CI = 1.14–4.04, p = 0.018/ OR = 3.45, 95% CI = 1.52–7.81, p = 0.003) and the subjective financial burden (OR = 2.54, 95% CI = 1.25–5.14, p = 0.010/OR = 3.89, 95% CI = 1.68–9.00, p = 0.002) were significantly related to withdrawal or change of cancer treatment (recommended by physicians/based on patient request). |
| (Aoyama et al., 2021) | To examine current financial status, changes before and after bereavement, and their effect on major depressive disorder and complicated grief among the bereaved family members of patients with cancer. | A total of 491 (62%) questionnaires were returned. Of those patients, the median age was 74.4 ± 11.5 years, and 53% were male. The average age of the bereaved family members was 62.5 ± 11.6 years, and 68% were female. | Cross-sectional, anonymous, and self-reported questionnaire survey, part of the Japan Hospice and Palliative care Evaluation study 2016: J-HOPE2016 study | Majority of the participants (78%) reported having no or mild concerns about their livelihood, whereas 19% had moderate to severe concerns. Regarding the change in financial status after bereavement, 7% reported improvement, 28% reported worsening, and 65% reported no change. The prevalence of a possible major depressive disorder and complicated grief were 22% and 9%, respectively, and were significantly lower among participants with less concern regarding their livelihood and whose financial status had not changed after the bereavement (both p < 0.05). |
| (Watanabe et al., 2021) | To evaluate the disease-specific, per-patient costs of the five major cancers using Japan’s cancer registry data and health service utilization data. | The study analyzed 304,698 patients with five major cancers - stomach, lung, colorectal, liver, and breast cancers. | Analysis of a nationwide hospital-based cancer registry and diagnosis procedure combination survey | Lung cancer had the highest median overall costs per patient (2,508,789 JPY), while breast cancer showed the lowest (1,559,274 JPY). Based on the reported clinical stage, stage III cancers exhibited the highest median inpatient costs, except for colorectal cancer, which had the highest inpatient cost at stage IV. Stage III cancers also showed the highest outpatient median costs, except for liver and colorectal cancers. According to age group, patients in their 60s and 70s had the highest cancer care costs for all cancers. However, unlike other cancers, more than 50% of breast cancer care costs were consumed by patients under 60 years old. The costs for all cancers, excluding breast cancer, were the highest in the first month after diagnosis, but in the second month, breast cancer had the highest cost. |
| (Kaneko et al., 2020) | To estimate the effect of cancer diagnosis on the labor-force participation among middle-aged and older populations in Japan. | Data from nationwide annual surveys of a targeted population aged 51–70, examined respondents' cancer diagnoses and whether they continued to work, while also considering differences between gender (observations: 53,373 for men and 44,027 for women) and occupation type (observations: 64,501 for cognitive workers and 20,921 for manual workers). | Nationwide population-based longitudinal survey | Male workers are more likely to quit their job the year they were diagnosed with cancer or in the following year. Female workers were more likely to quit their job immediately after being diagnosed with cancer. However, this effect totally disappears when considering likelihoods in the following year. Cognitive workers are more likely to quit their job in the year of diagnosis by 11.6%, and this effect remains significant at 3.8% in the following year. The effect during the year of diagnosis on manual workers is huge. It accounts for 18.7%; however, the effect almost disappears in the following year. |
| (Ohno et al., 2020) | To reveal the humanistic and economic burden among caregivers of cancer patients, and to compare it with the burden among caregivers of patients with other conditions (other caregivers) and non-caregivers. | A total of 251 caregivers of cancer patients, 1,543 other caregivers, and 27,300 non-caregivers were identified. Caregivers of cancer patients (average age 48.0 years) were younger than non-caregivers (51.5) and other caregivers (54.4) and had the highest education level (57.8% completed their university education). | Cross-sectional multivariate analysis（Data from the 2017 National Health Survey） | Caregivers of cancer patients were younger and had the highest education level. Caregivers of cancer patients had significantly lower mental component summary scores than non-caregivers (40.18 vs. 46.70), and the difference indicated a clinically meaningful decrease in HRQoL. Also, reported were higher presenteeism (37.31% vs. 20.43%), total work productivity impairment (38.85% vs. 21.98%), activity impairment (40.94% vs. 25.78%), and total indirect costs (36.34% vs. 20.03% of average annual income). |
| (Honda et al., 2019) | To report the burden and characteristics of financial toxicity among Japanese patients with cancer using COST. | Patients receiving ongoing chemotherapy at a public regional cancer center in Japan were recruited. Of the 191 patients, 156 (82%) responded to the questionnaire. The median age of the respondents was 67 years (ranging from 30 to 87 years), and 83 patients (53%) were men. Ninety-six patients (62%) were receiving treatment with at least one molecular targeted agent. No patients received immunotherapy. The median duration from the start of chemotherapy was 12 months (range, 2 to 138 months). The most frequent category of annual household income was between ¥2 million to ¥4 million, and the household savings was more than ¥15 million. Twenty-nine patients (19%) had to retire from their work because of cancer. | Cross-sectional survey by mailing self-administered questionnaires | Median COST score was 21 (range 0 to 41; mean ± standard deviation, 12.1 ± 8.45), with lower COST scores indicating more severe financial toxicity. The multivariable analyses using linear regression revealed that older age (*β*, 0.15 per year; 95% CI, 0.02 to 0.28; P=.02) and higher household savings (*β*, 8.24 per ¥15 million; 95% CI, 4.06 to 12.42; P<.001) were positively associated with the COST score. |
| (Taguchi et al., 2019) | To examine the percentage of patients with breast cancer who changed their job around the surgery. | Participant’s average age was 45.9 (SD= 6.2) years old (range 25**–**57), and those in their 40s occupied 57% of the total. Of those, 52% had received a mastectomy, and 34% had received multiple drug therapies (chemotherapy and hormone therapy). | Secondary analysis of data from the universal healthcare insurance system | Nineteen percent of patients changed their employment status before and after surgery. Of those, 19% changed their employment by the end of the surgery month, while 42% did by the fourth month after surgery. The type of surgery had a significant effect on employment change, and people with mastectomy were more likely to change jobs. Also, patients with multiple drug therapies tend to change jobs. There were no significant relationships between employment change and age or insurance type. |
| (Honda et al., 2018) | To report on the pilot phase of the study assessing the feasibility of using the Japanese version of the COST questionnaire in measuring financial toxicity among Japanese cancer patients. | Patients who were receiving ongoing chemotherapy were recruited. The media age of the 11 patients was 65 (range 30–72), with 8 (73%) being female. Eight patients (73%) received treatment with at least one molecular targeted agent. The median length of time from the start of chemotherapy was 9 months (range, 2–55 months). | Prospective pilot survey by mail to ascertain the feasibility of using the COST questionnaire | The median COST score was 22 (range, 6–29; mean ± SD, 20.18 ± 8.17). Five (45%) and two (18%) patients suffered grade 1 (COST score 14–25) and grade 2 (COST score 1–13) financial toxicity, respectively. The COST values demonstrated good internal consistency with a Cronbach *α* of 0.87. |
| (Oizumi et al., 2018) | To identify (1) the quality of life of family members having pancreatic cancer patients at the time of completion of surgical therapy or adjuvant chemotherapy and during chemotherapy, and (2) the relationship between family members' perception of the patient's symptoms, supportive care status, patient treatment status, and the quality of life of the family members. | Sixty-seven family members of pancreatic cancer patients were recruited. The average age was 60, with 75% female, and 52% were employed. | Cross-sectional, self-administered questionnaire survey | Compared to the adjusted national standard, "daily role functioning (mental)" was significantly lower (p < 0.05). In addition, "vitality," "daily role functioning (mental)," and "mental health" were significantly lower for those with higher total scores of patient symptoms as perceived by family members (r^2^ = -0.29 to -0.39; p < 0.01 to 0.05). In the comparison of QOL in the medical care support status, QOL was significantly lower for those who had dietary burdens owing to patient symptoms (p < 0.01 to 0.05) and for those who had financial burdens (p < 0.05). |
| (Tsuchiya et al., 2018) | To clarify (1) the level of recognition and use of public support systems and (2) the level of recognition and use of employment support services in cancer-specialized hospitals. | The median age at the time of the survey was 57 years (range 26**–**90 years), and 44.2% were male. The median age at the time of cancer diagnosis was 51 years (range 20**–**81 years). The full-time employees were 4.7%, while 20.7% were self-employed/free, 16.9% were part-time employees, 8.3% were temporary/contract employees, and 5.4% were categorized under others. | Cross-sectional, self-administered questionnaire survey | The proportion aware of the national Accident and Sickness Benefits was 12％, and those aware of individual consultation by labor and social security attorneys was 46％. Those who had independently searched for the national financial support systems represented 40％. |
| (Sugiyama et al., 2017) | To identify factors affecting the quality of life of family caregivers who had cancer patients by using the Japanese version of the caregiver quality of life index-cancer (CQOLC) | The mean age of family caregivers was 48 ± 12 years, and 50% were male. The caregivers were children (53%), spouses (24%), and parents (14%). The most common employment status was full-time (57%). Thirty percent of the caregivers had a change in employment after the patient became ill, and their income status remained unchanged (53%) or decreased (45%). Regarding patients, the mean age was 71 ± 15 years, 51% were male, and 28% had been diagnosed for more than 5 years, 18% for less than 1 year, 16% for 1 to 2 years, and 16% for 2 to 3 years. Those undergoing periodic examinations after treatment was 32%, 27% were undergoing treatment for the disease itself, and 23% were undergoing treatment to alleviate symptoms. The patients' activity status (performance status) was 0-1 in 40%, 2-3 in 46%, and 4 in 15%. | Cross-sectional and internet survey | A univariate analysis of the economic burden domain of the CQOLC showed the following factors were associated with the caregiver’s burden: caregiver was younger (p < 0.01), household income was lower (p < 0.01), marital status was separated (p= 0.03), caregiver had a change in employment (p < 0.01), working hours were reduced (p= 0.02), resigned from his/her job (p= 0.04), income has decreased since the patient became ill (p < 0.01), there are other family members who need care (p= 0.03), the caregiver does not think the relationship with the patient is good (p < 0.01), the patient is younger (p < 0.01). |
| (Takura et al., 2016) | To report factors that influence the opinions of oncologists on health economics in cancer treatment and to evaluate the influence of the physician’s experience on the patient’s access to treatment technologies and the maximum public medical expenses associated with treatment. | To take part in this study, an oncologist must (1) achieve accountability for standard medical procedures in all phases of cancer treatment; (2) possess the knowledge and experience required of a certified doctor; (3) be able to understand state-of-the-art technology in specialized areas such as surgery, medication, and radiation; and (4) be a doctor or dentist. The number of effective responses was 172. The mean number of postgraduate years was 30.3 ± 6.2 years, and the mean number of patients seen was 1323 ± 1963 cases/year. Many of the respondents were hospital doctors who specialized in treating digestive organs (n = 66, 38.5 %), followed by urologists (n = 24, 14.0 %), other general surgery doctors (n = 22, 12.8 %), gynecologists (n = 13, 7.6 %), and medical oncologists (n = 11, 6.4 %). | Secondary analysis of the cross-sectional survey, JSCO | Of 172 respondents, 66 (41.0 %) believed that the maximum allowable medical expenses for cancer treatment should be ≤4 million yen/LY, while 62 (39.8 %) reported a value of 4.01–8 million yen/LY (LY: life year). |
| (Ito et al., 2015) | To examine the effect of demographic, clinical, and employment characteristics on the return to work for newly diagnosed cancer patients in Japan, with a particular emphasis on non-regular employees. | The mean age at diagnosis was 54.9 years, and the average number of years after diagnosis was 4.2 years. Most patients (65.0%) reported that their health status was fair. Almost 74.5% of the patients had surgery, 65.7% had chemotherapy, and 37.5% had radiation therapy. Half (48.8%) of the patients were regular employees, 26.0% were self-employed, 15.7% were non-regular employees, and 9.4% were regular employees working in the public sector at the time of diagnosis. At ≥1 year after cancer diagnosis, 24.2% of patients were not employed at the same work or at any other work. | Cross-sectional, self-administered questionnaire survey | A high proportion of patients (75.8%) had returned to work a few years after a cancer diagnosis. Non-regularly employed survivors were less likely to return to work. Individuals with poor health, advanced-stage tumors, older age, and women were significantly less likely to return to work. Only 52.8% of non-regular employees continued to be employed, and their income decreased by as much as 61.1%. |
| (Okada et al., 2015) | To investigate the following issues and relevant changes for the working mothers of children diagnosed with cancer: (1) work change, (2) stress, (3) social support, (4) work motivation, and (5) employment status after diagnosis. | Of the 62 mothers, approximately half were 40 to 49 years old, and 6.9% were single parents. Of those who had jobs during the diagnosis, 8 (25.0%) were the primary wage earners for the family. Twenty-seven (46.5%) had to stay 24 hours a day with their children at the hospital. | Cross-sectional exploratory study | Of the 32 mothers who worked at the time of diagnosis, 10 continued to work, 12 took an extended leave, and 10 quit their jobs, and 70% lost motivation for work following the diagnosis. Half of mothers who continued to work during treatment reported financial reasons. No significant differences in demographic data, including age of the mothers, their level of education, their change of residence, and whether they stayed 24 hours a day with their children at the hospital, were observed between the change in employment group and the no-change in employment group. |
| (Umezawa et al., 2015) | To describe cancer survivors’ supportive care needs in Japan, identify associated factors of unmet needs, and describe the sources of support that are preferred and actually used by cancer survivors. | Of the 628 respondents who completed the questionnaire, 46 were excluded due to missing values. The participants' mean age was 56 years, and most were in good performance status. The type of cancer was skewed towards breast and prostate cancers, and the proportion of lung and gastric cancers were smaller than the Japanese general population sample. | Secondary analysis of cross-sectional web-based survey | The prevalence of unmet needs ranged from 5 to 18%, depending on different domains. The prevalence was high in the financial and medical-psychological domains and relatively low in the physical and sexual domains. Lower income was associated with unmet medical-psychological, financial, and social-spiritual needs. The prevalence of unmet needs did not differ among groups in terms of timing. |
| (Saito et al., 2014) | To examine the impact of breast cancer on the work-related lives of Japanese women and identify factors that correlate with job resignation. | All respondents were female (mean age at diagnosis, 42.5 ± 6.4 years; median time since diagnosis, 40 months; range, 1**–**185 months). At the time of diagnosis, 24 respondents (22.9%) had dependent family members, 68 (64.8%) were full time workers, 34 (32.4%) had access to an occupational health physician at their workplace, 47 (44.8%) were specialists and technical workers, and 43 (40.9%) were clerical workers. | Cross-sectional internet survey | Thirty-one respondents (29.5%) lost their jobs, and 12 could not find another job after breast cancer diagnosis. Nearly half of the respondents (47.6%) reported a decrease in personal income after diagnosis. Contract and part-time workers were significantly more likely to lose their jobs compared to regular, full-time workers. Seventy-nine respondents (75.2%) consulted someone regarding work-related issues. The most frequently consulted person was their boss at work. |
| (Okubo, 2013) | To identify the difficulties spouses of patients diagnosed with cancer face during the recuperation process from the time the patient is diagnosed with cancer to end-of-life care. | Eight spouses of patients who died of cancer in their prime of life or in old age participated in this study. The ages of the spouses ranged from 49 to 79 years (mean age 67 ± 7 years), one male and seven females. The length of time since bereavement varied from about 3 months to about 24 years. | A qualitative inductive study using semi-constructive interviews | Difficulties related to cancer treatment faced by the spouses of cancer patients included treatment decisions, agreement with the patient regarding treatment options, and the financial burden of treatment. The two components of financial burden were 1) the high cost of medical care and 2) changes in lifestyle patterns that weigh heavily on financial issues. |
| (Kodama et al., 2012) | To evaluate the hypothesis that patients’ income and the financial burden of their treatment could influence their willingness to undergo such medical interventions, especially on chronic myelogenous leukemia (CML) patients who have been prescribed imatinib. | We asked 345 hospitals across Japan with haematology departments to participate in our survey. Of them, 144 (41.7 %) agreed to participate, and the questionnaires were distributed to 1200 CML patients. The median age was 61 years (range, 15**–**94), and 428 patients (74.4 %) were ≤ 70 years old. | Cross-sectional survey by mailing self-administered questionnaires | A financial burden was felt by 41.2 % of the patients in 2000, 70.8 % in 2005, and 75.8 % in 2008. Overall, 31.7 % considered discontinuation because of the financial burden, and 2.6 % temporarily stopped their imatinib prescription. Older patients (OR = 0.96, 95 % CI: 0.95–0.98, p < 0.0001 for 1-year increments), and patients with higher household incomes (OR = 0.92, 95 % CI: 0.85–0.99, p = 0.03 for 10,000 USD-increments) were less likely to have considered discontinuing their imatinib treatment. |
| (Osono et al., 2012) | To clarify problems and their solutions regarding home care for terminal cancer patients from the perspective of home health care nurses based on their interviews. | The number of full-time nurses working at the home-visit nursing station was 25 (Chiba: 7, Kanagawa: 7, Tokyo: 6, Saitama: 3, and Ibaraki: 2), and about half of them had more than 10 years of experience as home-visit nurses. Half of the participants, involved in more than 10 cases with cancer patients in the past year, were assigned to the study. Another 19 participants were qualified as care managers. | Qualitative inductive research | The problem of the financial burden on home care users included the following two items: "If long-term care insurance is not available, the patient bears 30% of the cost, which is a heavy financial burden" and "The increase in revenue of long-term care agencies leads to an increase in the patient's out-of-pocket expenses, and there is a contradiction between the revenue and the provision of care.”  Solutions to these problems are to "evaluate the content and the price of those services with patients and families and consider the balance between the number of services and the financial burden" and "consider together whether there are ways to reduce the financial burden". The importance of explaining the necessity of medical and welfare services while working together to reduce the financial burden was mentioned. |
| (Komura et al., 2011) | To classify the desires of patients and bereaved families by analyzing the answers to the open-ended questions in the questionnaire survey conducted as a preliminary study, and identify areas for improvement needed in the future. | From the 1,493 advanced cancer patients and 1,658 bereaved families in four areas who received the questionnaire, 271 patients and 550 families filled in the free description. | Cross-sectional survey, self-administered questionnaire survey by mail (The Outreach Palliative Care Trial of Integrated Regional Model (OPTIM) study) | The most common request from patients was for improvements in the medical system (50.2%), with "reducing the financial burden" (10.3%) being the most common, followed by "improving the in-hospital system" (8.1%). Other opinions included "provide information about hospice and palliative care to the general public" (10.7%) and "provide adequate pain relief" (9.6%). |
| (Mitsuki et al., 2010) | To identify concerns and preferred support of cancer patients and their families who have transitioned from a cancer treatment center to outpatient services. | Regarding the patients’ backgrounds, 58.9% (n= 95) and 71.7% (n= 53) were male, and 35.8% and 34.0% were in their 60s and 70s at the prefectural base hospitals and regional base hospitals, respectively. The most common current treatment was chemotherapy (69.8%) at the regional base hospitals, while 48.5% and 44.2% received surgery and chemotherapy, respectively, at the prefectural base hospitals. The female proportion of patients' family members was 76.4% and 87.0% at the prefectural base hospitals and regional base hospitals, respectively. | Cross-sectional survey, self-administered questionnaire survey by mail | Prognosis was the most common concern for patients (78.4%), followed by symptoms (24.3%), financial burden (20.3%), work (20.3%), treatment (19.6%), family (18.9%), death (12.8%), personal care (10.8%), and relationship with doctors (7.4%).  Family members were concerned about the prognosis (87.3%), symptoms (33.3%), treatment (29.4%), financial burden (23.0%), death (14.3%), work (11.9%), relationship with medical personnel (8.7%), personal care (5.6%), and family (4.7%). |
| (Hayashida et al., 2005) | To identify difficulties and coping strategies by interviewing cancer patients living with chemotherapy in an outpatient setting. | Eight women with recurrent or metastatic breast cancer participated, mean age was 53.3 years, and their Performance Status were 0-1. | Qualitative inductive research | Difficulties arose because cancer patients had to pay high costs each time they received treatment. The three main categories included: significant cost of treatment (because treatments were not covered by insurance), high cost of drugs, and family finances being squeezed by the cost of treatment. |
